# Sarcoidosis With Multiple Bone Lesions Mimicking Advanced Lung Cancer With Multiple Bone Metastases

**DOI:** 10.7759/cureus.56915

**Published:** 2024-03-25

**Authors:** Takuya Ogaito, Yukio Kawagishi, Atsushi Muto, Akihiro Kikushima

**Affiliations:** 1 Department of Respiratory Medicine, Kurobe City Hospital, Kurobe, JPN

**Keywords:** bone lesion, sarcoidosis, positron emission tomography, lung cancer, bone metastases

## Abstract

Bone lesions in sarcoidosis are more common than previously known. A 59-year-old female with a history of sarcoidosis was referred due to suspected lung cancer. 18F-fluorodeoxyglucose positron emission tomography/computed tomography (PET/CT) revealed numerous bone lesions in addition to abnormal uptake by pulmonary nodules and mediastinal lymph nodes, which mimicked metastatic advanced lung cancer. Biopsy of bone lesions detected epithelioid cell granuloma consistent with bone sarcoidosis. Moreover, prednisolone treatment was tried to exclude malignant disease. One month after prednisolone administration, bone lesions and other abnormal uptake disappeared on PET/CT. Bone sarcoidosis is often asymptomatic and is discovered incidentally as multiple lesions that may require differentiation from malignant disease. Biopsy of bone lesions and administration of corticosteroids may be useful for accurate diagnosis.

## Introduction

Sarcoidosis is a systemic inflammatory disease involving multiple organs. Although osseous involvement is a less common manifestation of sarcoidosis (3.4%), it has been detected more often with the use of magnetic resonance imaging and 18F-fluorodeoxyglucose positron emission tomography/computed tomography (PET/CT) for diagnosis [1]. Nonspecific pain is the most common feature of axial skeletal sarcoidosis; however, half of patients are asymptomatic [1]. Sarcoidosis with multiple bone lesions requires differentiation from metastatic bone tumors. Herein, we report a case of sarcoidosis with extensive bone lesions that required differentiation from a metastatic malignant lung tumor.

## Case presentation

**Figure 1:**
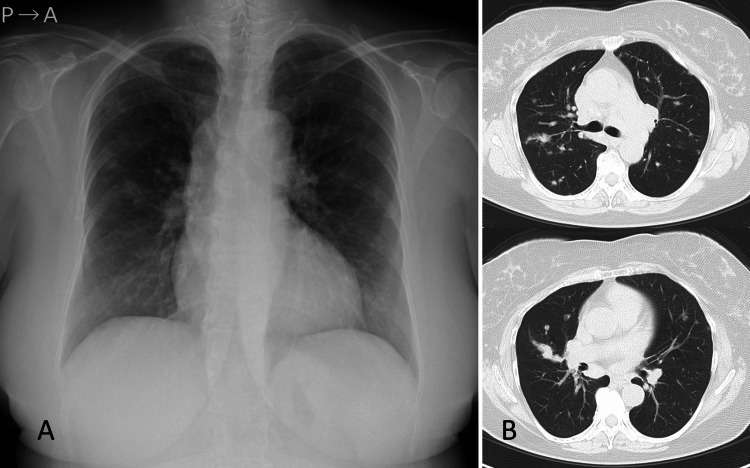
Chest images (A) Chest radiograph showing a new nodular lesion in the middle of the right lung. (B) Computed tomography scan of the chest revealing a new pulmonary nodule (2.5 cm in diameter) within the right superior lobe, multiple smaller nodules that are predominantly distributed in the superior lung fields, and enlarged mediastinal lymph nodes.

A 59-year-old female who had never smoked was referred to our hospital for a nodular shadow in the right lung on a chest radiograph that was performed during a routine medical check-up (Figure 1A).

**Figure 2:**
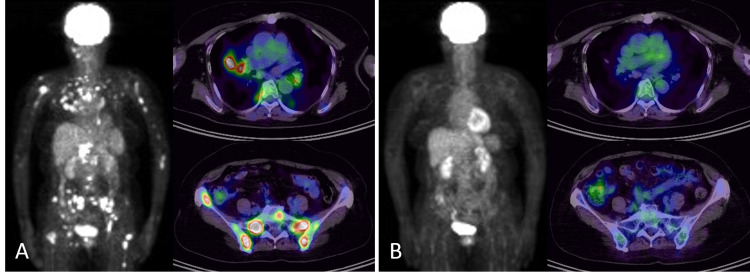
An 18F-fluorodeoxyglucose PET/CT (A) An 18F-fluorodeoxyglucose PET/CT scan showing increased uptake of fluorodeoxyglucose by the mediastinum, nodular lung lesions (SUV: 7.08 (a nodule in the right upper lobe)), abdominal lymph nodes (SUV: 13.38), and foci in multiple bones, including the axial skeleton and pelvis (SUV: 8.08 (the left sacrum)). (B) Four weeks after the start of prednisolone, showing no abnormal uptakes on PET/CT. PET/CT, positron emission tomography/computed tomography; SUV, standardized uptake value

The patient had experienced mild back pain for several months and had been diagnosed with sarcoidosis nine years previously. Bilateral hilar lymphadenopathy and pulmonary lesions of sarcoidosis had not worsened during three years of follow-up. Physical examination showed no abnormalities. Laboratory data were normal, except for slight increases in soluble interleukin 2 receptor and lysozyme (887 U/mL). Chest CT scan revealed a new pulmonary nodule (2.5 cm in diameter) in the right superior lobe, multiple smaller nodules in the bilateral lungs, and enlarged hilar and mediastinal lymph nodes (Figure 1B). Although the multiple pulmonary nodules and enlarged lymph nodes were seen in the previous CT scan, these structures had increased in size. PET/CT was positive for multiple pulmonary nodules, the new larger nodule, swollen lymph nodes, and numerous foci within bones of the axial skeleton and pelvis (Figure 2A).

**Figure 3:**
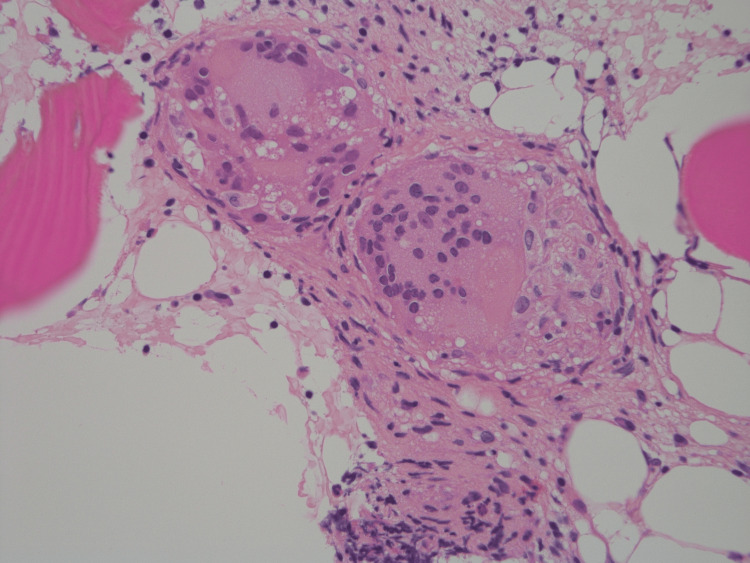
Bone biopsy A bone biopsy of the ilium revealing a non-caseating epithelioid granuloma (H&E stain)

T2-weighted pelvic magnetic resonance imaging showed high-intensity signals within the ilium and sacrum that corresponded with positive foci on PET/CT. These findings closely mimicked advanced lung cancer with multiple bone metastases. However, the patient’s overall condition seemed inconsistent with advanced metastatic lung cancer. Bronchoscopic biopsy showed no evidence of malignancy. Bone biopsy of the ilium revealed epithelioid cell granulomas consistent with sarcoidosis (Figure 3).

Although the patient’s symptoms were mild, developing skeletal impairment was a concern. Prednisolone 30 mg/day (0.5 mg/kg) was administered to rule out possible malignancy. One month later, the patient’s back pain had improved, and a chest CT scan showed shrinkage of the multiple pulmonary nodules and swollen lymph nodes. PET/CT showed the disappearance of abnormal findings (Figure 2B). Prednisolone was gradually tapered to 2 mg/day and no relapse occurred without any fractures for three years.

## Discussion

Bone sarcoidosis is characterized by sarcoid granulomas in the bone marrow that are identical to those in other organs, which sometimes present with local pain, but are often asymptomatic and incidentally detected on imaging [1-3]. Thus, the occurrence of bone sarcoidosis may be underestimated. With the widespread use of PET/CT and other imaging techniques, bone sarcoidosis is now more often detected and usually involves multiple lesions in the axial skeleton and other bones [1]. Many of the sarcoidosis bone lesion sites that predominate in the axial skeleton overlap with sites that are characteristic of bone metastases of cancer. If PET/CT demonstrates such bone lesions in a patient with suspected malignancy, the findings are often mistaken for bone metastases, even if the patient has a history of sarcoidosis. The standardized uptake values of neoplastic lesions and granulomas of sarcoidosis are similarly high in lymphadenopathy, pulmonary lesions, and bone lesions; thus, it is difficult to distinguish between the two by PET/CT [4]. The PET/CT findings in our patient with a new pulmonary nodule resembled advanced metastatic lung cancer with multiple bone metastases. However, her overall good condition was inconsistent with metastatic lung cancer.

Case reports of bone sarcoidosis that require differentiation from malignant diseases have increased in recent years. Reported cases involved numerous bone lesions, a history of malignancy such as breast cancer, and high levels of soluble Interleukin-2, which can be caused by lymphoma [4,5]. Most sarcoidosis cases with bone involvement affect the spine or pelvis and are often asymptomatic, although cases with pain, neurological symptoms, and pathological fractures have also been reported [2,5,6]. In most instances, bone biopsies revealed noncaseous epithelioid granulomas consistent with sarcoidosis, and corticosteroid treatment usually improved the lesions as well as the granulomas of the other organs [2,3,6].

Sarcoidosis patients have an increased risk of many malignant diseases including lung cancer and lymphoma, which are presumably influenced by chronic inflammation, immune dysfunction, shared etiologic agents, genetic susceptibility, and induction by some drugs. Furthermore, a sarcoid-like reaction to a tumor (tumor-related granulomatosis) is difficult to distinguish from sarcoidosis [7]. Therefore, accurate diagnosis of sarcoidosis depends not only on compatible clinical presentation but also on distinctive histopathological findings and exclusion of differential diagnoses. Since bone sarcoidosis mimics bone metastases, the exclusion of malignant disease is critical in the diagnosis and management of sarcoidosis.

Bone sarcoidosis can induce pathological fractures; however, we could not find any reports of corticosteroid treatment inducing fractures in bone lesions. Although there is no consensus on the duration of corticosteroid treatment and long-term treatment leads to new fracture risks, corticosteroid treatment may be reasonable for symptomatic patients and for the prevention of pathological fractures at bone lesions. It is also one option for ruling out bone metastases. The induction and duration of corticosteroid treatment should be allowed to be clinically determined.

## Conclusions

PET/CT findings for multiple bone lesions of sarcoidosis can mimic those of bone metastases of malignant tumors, requiring differentiation. Biopsy of the bone lesion is essential for accurate diagnosis, and administration of corticosteroids may be helpful for confirming the diagnosis.
